# 

**Published:** 1903-05

**Authors:** 


### CONTENTS.

ORIGINAL ARTICLES:
President's Address. By W. L. Rhoads, D.V.S. . . . 269
Modes of Tubercular Infection in Wild Animals in Captivity. By W. Reid Blair, D.V.S. 278
Acute Pleurisy in Horses. By Prof. A. H. Baker . . . 281
Intestinal Antisepsis. By J. H. Crawford . . . 284
Sclerostoma Tetracanthus. By Dr. N. I. Stringer . . . 287
Analogous Foot-and-Mouth Disease. By C. C. Mills, V.S. . . . 292
Gastro-Intestinal Catarrh of the Ox By Dr. F. H. Barr . . . 296
Azoturia. By F. H. Ames, D.V.S. . . . 298

SELECTIONS:
Actinobacillosis. By S. J. J. Harger, V.M.D. . . . 300
Notes on Arab Horses. Contributed by the Rev. Franklin E. Hoskins . . . 303

REPORTS OF CASES:
A Case of Milk-Fever. A New Treatment. By E. H. Lehnert, D.V.S. . . . 307
Lipoma of a Horse. By John J. Repp, V.M.D. . . . 310

EDITORIALS: Canada's Peril—College Changes . . . 312
LEGISLATION: New Jersey—Conviction Under New Jersey's New Veterinary Law. Warning to Offenders—The Effect of the New Jersey Law . . . 313
AMONG THE COLLEGES . . . 314
COMMENCEMENT EXERCISES: Postgraduate Course, Chicago Veterinary College—Chicago Veterinary College Banquet—Chicago Veterinary College . . . 315–317
SOCIETY PROCEEDINGS—A. V. M. A.—PERSONALS . . . 320–335

